# Detection of QTNs for kernel moisture concentration and kernel dehydration rate before physiological maturity in maize using multi-locus GWAS

**DOI:** 10.1038/s41598-020-80391-1

**Published:** 2021-01-19

**Authors:** Shufang Li, Chunxiao Zhang, Deguang Yang, Ming Lu, Yiliang Qian, Fengxue Jin, Xueyan Liu, Yu Wang, Wenguo Liu, Xiaohui Li

**Affiliations:** 1 Crop Germplasm Resources Institute, Jilin Academy of Agricultural Sciences, Kemaoxi Street 303, Gongzhuling, 136100 Jilin Province China; 2 College of Agronomy, Northeast Agricultural University, Harbin, 150030 China; 3 Maize Research Institute, Jilin Academy of Agricultural Sciences, Gongzhuling, 136100 China; 4 Maize Research Center, Anhui Academy of Agricultural Science, Hefei, 230001 China; 5 Gongzhuling Meteorological Bureau, Gongzhuling, 136100 China

**Keywords:** Agricultural genetics, Plant breeding, Plant genetics

## Abstract

Maize is China’s largest grain crop. Mechanical grain harvesting is the key technology in maize production, and the kernel moisture concentration (KMC) is the main controlling factor in mechanical maize harvesting in China. The kernel dehydration rate (KDR) is closely related to the KMC. Thus, it is important to conduct genome-wide association studies (GWAS) of the KMC and KDR in maize, detect relevant quantitative trait nucleotides (QTNs), and mine relevant candidate genes. Here, 132 maize inbred lines were used to measure the KMC every 5 days from 10 to 40 days after pollination (DAP) in order to calculate the KDR. These lines were genotyped using a maize 55K single-nucleotide polymorphism array. QTNs for the KMC and KDR were detected based on five methods (mrMLM, FASTmrMLM, FASTmrEMMA, pLARmEB, and ISIS EM-BLASSO) in the package mrMLM. A total of 334 significant QTNs were found for both the KMC and KDR, including 175 QTNs unique to the KMC and 178 QTNs unique to the KDR; 116 and 58 QTNs were detected among the 334 QTNs by two and more than two methods, respectively; and 9 and 5 QTNs among 58 QTNs were detected in 2 and 3 years, respectively. A significant enrichment in cellular component was revealed by Gene Ontology enrichment analysis of candidate genes in the intervals adjacent to the 14 QTNs and this category contained five genes. The information provided in this study may be useful for further mining of genes associated with the KMC and KDR in maize.

## Introduction

Maize (*Zea mays* L.) is the largest grain crop in China. The planting area for maize and maize production were 41.3 million ha and 2.6 billion tons in 2019 in China, respectively^1^. Mechanical grain harvesting is the key technology in maize production, and the kernel moisture concentration (KMC) is the main controlling factor in mechanical maize harvesting in China^2^. A low KMC at harvest may facilitate mechanical harvesting, shelling efficiency, and grain quality and reduce additional drying cost and shrinkage penalties^3–5^. When the KMC at harvest is greater than 25 percent, the breakage rate quickly increases, which significantly reduces farmers' incomes^6^. The kernel dehydration rate (KDR) is defined as the rate of moisture loss between two adjacent periods after pollination, which is closely related to the KMC. It is the key measurements to select maize hybrids with a low KMC and a high KDR at mature period for achieving mechanical maize harvesting.

Changes in the KMC and KDR occur in two distinct phases^7,8^. The first phase spans the time from pollination to physiological maturity (PM) and is defined as physiological dehydration. The second phase spans the time from PM to harvest and is defined as a natural drying process. During the first phase, kernel dehydration is an internal process under the control of growth and development regulatory processes. Environmental factors have no significant effects on such dehydration^9^. Beginning as early as the 1960s, the KMC and KDR have been controlled by multi-genes and could be stably inherited^10–12^. Previous research has also shown that selection based on a low KMC and a high KDR before PM was an effective strategy to achieve a low KMC at harvest^13–15^. Therefore, measuring the KMC and KDR in various maize cultivars before PM and conducting quantitative trait locus (QTL) mapping, genome-wide association studies (GWAS), and candidate gene mining for these two traits in maize are crucial tasks.

Extensive research has been conducted to map QTLs for the KMC and KDR in maize^3,4,16–19^. Some intervals or genes associated with the KDR in maize have been obtained^3,4^, but only Dai et al.^20^ and Zhang et al.^21^ have conducted GWAS for these two traits in maize.

The most popular method for GWAS is the mixed linear model (MLM)^22,23^. In the past decade, several MLM algorithms, such as compressed MLM^24^ and enriched compressed MLM^25^, were developed to improve the computational efficiency. However, all these models perform one-dimensional genome scans and require multiple corrections. Generally, the above traditional methods had significant limitations in mapping QTLs with relatively small effects. Therefore, Wang et al.^26^ proposed a new model. Then, GWAS methods based on a multi-locus random-SNP-effect MLM (mrMLM) were proposed. The methods included iterative modified-sure independence screening expectation–maximization (EM)-Bayesian LASSO (ISIS EM-BLASSO), polygenic-background-control-based least angle regression plus empirical Bayes (pLARmEB), fast multi-locus random-SNP-effect efficient mixed model association (FASTmrEMMA), and fast multi-locus random-SNP-effect mixed linear model (FASTmrMLM)^26–30^. These methods could effectively detect small-effect quantitative trait nucleotides (QTNs) and improve the efficiency and accuracy of GWAS.

In this study, the mrMLM was used to perform a GWAS based on Axiom Maize 55K SNP Array data from 132 maize inbred lines. QTNs associated with the KMC and KDR were detected using five methods in the package mrMLM (mrMLM, FASTmrMLM, FASTmrEMMA, pLARmEB, and ISIS EM-BLASSO). Associated candidate genes were predicted and submitted to Gene Ontology (GO) enrichment analysis. The results have theoretical significance and application value for improving maize kernel dehydration characteristics using marker-assisted selection (MAS).

## Results

### Phenotypic data analysis of 132 inbred lines

**Figure 1:**
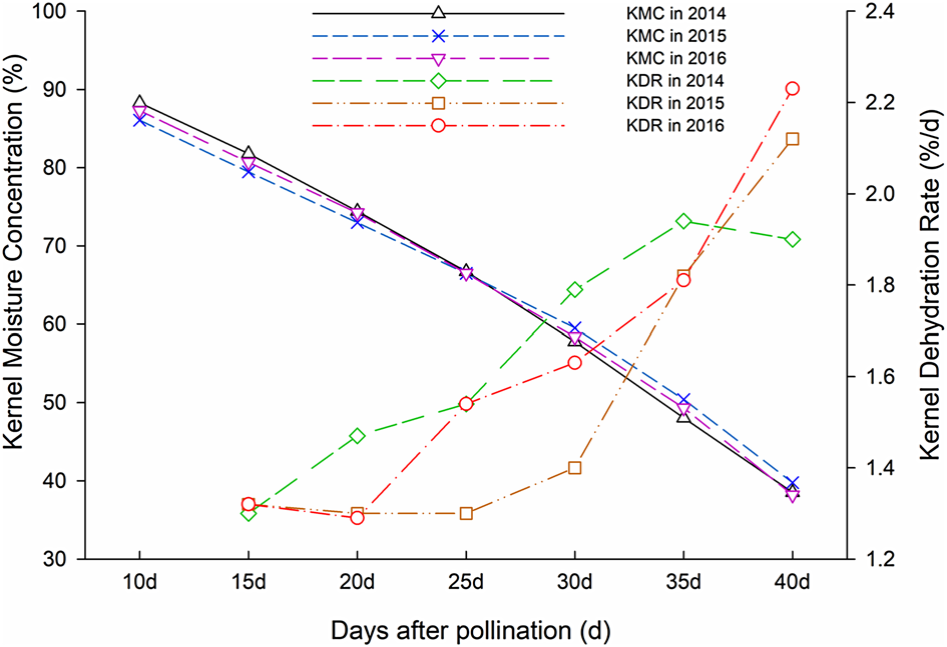
The changes of the KMCs and KDRs in 2014, 2015 and 2016 across time.

The trends of both the KMC and KDR were very similar in 2014, 2015 and 2016 (Fig. 1). The KMC gradually decreased over time, while the KDR gradually increased over time (Table 1). The different phenotypic traits also showed different levels of variation. The KMC had relatively low coefficients of variation (CV), and the lowest CVs were found at 15 days after pollination (DAP), with values of 1.34%, 1.48%, and 1.44% in 2014, 2015, and 2016, respectively. CVs for the KMC gradually increased over time, with values of 12.68%, 13.56% and 13.71% in 2014, 2015, and 2016 at 40 DAP, respectively. By contrast, the KDR had relatively high CV, with the maximum values found at 35 DAP, 42.09%, 39.4% and 37.06% in 2014, 2015, and 2016, respectively. This result suggests that the optimal time for maize KDR measurement may be after 35 DAP. The heritability ranged from 68.54 to 76.42% for the KMC, and ranged from 61.75 to 74.16% for the KDR. The highest heritability of both traits appeared at 40 DAP. The results from analysis of variance for maize KMC and KDR revealed highly significant differences across cultivars and across periods, except KDR15.

**Table 1:** Statistical analysis for the KMCs and KDRs of the 132 inbred lines.

Traits	Year	Max	Min	Mean	SD	CV (%)	Her. (%)	F_G_	F_E_
KMC10	2014	91.76	85.17	88.30	1.47	1.67	75.61	12.12**	325.38**
	2015	89.71	82.88	86.06	1.47	1.71			
	2016	91.4	83.81	87.28	1.48	1.70			
KMC15	2014	84.83	79.09	81.79	1.10	1.34	76.42	6.86**	330.23**
	2015	82.65	75.94	79.46	1.18	1.48			
	2016	83.13	77.65	80.69	1.16	1.44			
KMC20	2014	76.94	72.21	74.42	0.98	1.32	68.54	5.56**	135.57**
	2015	75.38	69.64	72.98	1.11	1.53			
	2016	77.18	71.28	74.23	1.12	1.51			
KMC25	2014	69.61	63.48	66.72	1.12	1.68	67.57	5.25**	3.03*
	2015	69.37	62.93	66.48	1.20	1.80			
	2016	69.00	63.71	66.50	1.14	1.72			
KMC30	2014	60.81	48.60	57.76	1.56	2.70	72.21	7.86**	99.23**
	2015	62.77	51.27	59.50	1.80	3.02			
	2016	61.54	49.52	58.36	1.66	2.84			
KMC35	2014	51.71	37.83	48.05	2.48	5.17	73.95	10.55**	79.57**
	2015	55.03	37.3	50.40	3.20	6.34			
	2016	53.79	36.75	49.31	2.95	5.99			
KMC40	2014	46.58	26.23	38.53	4.89	12.68	76.70	19.04**	22.57**
	2015	49.58	23.55	39.77	5.39	13.56			
	2016	47.32	22.72	38.18	5.24	13.71			
KDR15	2014	1.91	1.03	1.30	0.20	15.68	70.55	10.48**	1.20
	2015	1.80	1.04	1.32	0.20	14.91			
	2016	1.90	1.04	1.32	0.20	15.27			
KDR20	2014	2.00	1.31	1.47	0.13	9.02	62.92	9.64**	272.54**
	2015	1.93	1.10	1.30	0.14	11.18			
	2016	1.90	1.12	1.29	0.15	11.22			
KDR25	2014	2.30	1.39	1.54	0.12	7.87	61.75	6.57**	421.09**
	2015	2.09	1.11	1.30	0.14	10.57			
	2016	2.31	1.39	1.54	0.12	8.01			
KDR30	2014	3.23	1.62	1.79	0.18	10.27	66.55	10.20**	507.08**
	2015	2.79	1.20	1.40	0.21	14.91			
	2016	3.16	1.43	1.63	0.19	11.96			
KDR35	2014	3.44	1.62	1.94	0.34	17.29	68.16	12.95**	21.76**
	2015	3.70	1.40	1.82	0.43	23.45			
	2016	3.81	1.40	1.81	0.40	22.19			
KDR40	2014	4.20	0.85	1.90	0.80	42.09	74.16	36.67**	67.25**
	2015	4.67	0.95	2.12	0.84	39.40			
	2016	4.78	1.14	2.23	0.83	37.06			

*Max.* maximum; *Min.* minimum; *SD* standard deviation; *CV* coefficient of variation; *Her.* heritability; *F*_*G*_* and*^*7*^*F*_*E*_ the F-values for genotypes and environments, respectively.

* and **Denote significance at 0.05 and 0.01 levels, respectively.

### Population structure and linkage disequilibrium (LD) analysis

The Axiom Maize 55K SNP Array (CapitalBio Corp., Beijing, China) had 55,229 SNPs^31^, and the 132 inbred lines were genotyped based on the Affy AXIOM Array 2.0 platform. The raw genotyping data were filtered using PLINK based on a minor allele frequency (MAF) > 0.05 and a missing genotype rate (GENO) < 0.1. After filtering, the remaining 41,357 SNPs were used for LD analysis and GWAS.

**Figure 2:**
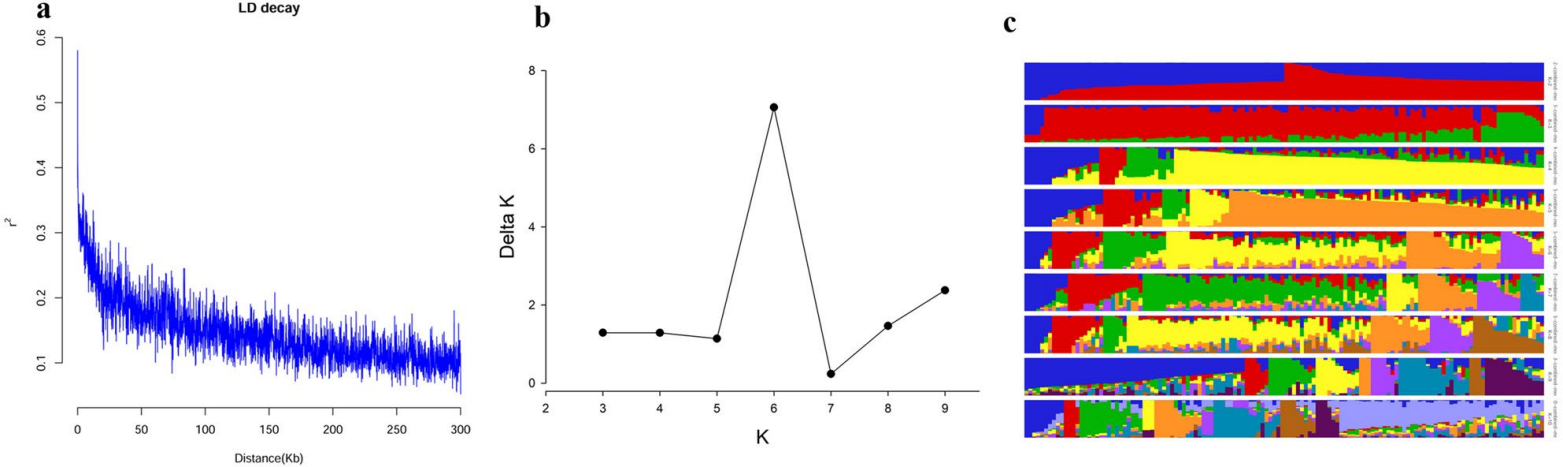
Linkage disequilibrium decay and population structure of 132 lines. (**a**) LD decay; (**b**) Plot of delta *K* against putative *K* ranging from 2 to 10; (**c**) Stacked bar plot.

When r^2^ = 0.1, the attenuation distance of LD was approximately 200 kb (Fig. 2A), and genes within 200 kb upstream or downstream of the marker exceeding the threshold were taken as candidates. The number of subpopulations (*k*) was plotted based on the delta k calculated using STRUCTURE software (Fig. 2B). The line chart showed a peak at *k* = 6, indicating that the natural population could be divided into six subpopulations (Fig. 2C).

### The quality and accuracy of the SNP markers and lines

Measurement of the KDR is relatively difficult, and natural populations have rarely been used in GWAS. In this study, we conducted the GWAS using cob colour, a qualitative trait in maize, to verify the validity and accuracy of the experimental populations and markers. Pericarp colour 1 (P1) regulates red pigmentation in cob, pericarp, tassel glumes, and husks, and its gene is located at mk187 on chromosome (chr.) 1 at position 48.1 Mb. A total of seven significant QTNs were detected on chr.1 using the package mrMLM (Table 2). Among them, AX-86284808 and AX-91425354 were detected by two and four methods, respectively, which explained up to 32.12% of the phenotypic variation. When r^2^ = 0.1 and the attenuation distance of LD was approximately 200 kb, AX-86284808 and AX-91425354 were mapped onto B73 Ref Gen_V4, and both QTNs were internally scanned to Zm00001d028850 (P1), which is consistent with the results of a previous study screening genes involved in cob colour^32^. These results suggest that we should identify candidate genes within the attenuation distance of LD around the QTNs shared in common by multiple detection methods to achieve accurate candidate gene mining (Table 2).

**Table 2:** QTNs for cob colour traits by multi-locus GWAS methods.

^a^SNP	Chr	Pos. (Mbp)	QTN effect	LOD score	PVE (%)	Method
AX-86239441	1	32.84	− 0.25	5.55	2.15	pLARmEB
AX-86239485	1	38.14	0.18	3.71	2.46	FASTmrMLM
AX-123944403	1	44.17	− 0.20	3.77	2.06	pLARmEB
AX-123945886	1	47.51	− 0.27	3.02	4.40	FASTmrMLM
AX-91462352	1	47.81	0.35	7.97	6.98	pLARmEB
**AX-86284808**	1	47.99	0.53	3.66	5.24	FASTmrEMMA
**AX-86284808**	1	47.99	0.25	5.05	4.72	ISIS EM-BLASSO
**AX-91425354**	1	48.00	1.09	11.39	22.33	FASTmrEMMA
**AX-91425354**	1	48.00	0.43	10.23	14.28	ISIS EM-BLASSO
**AX-91425354**	1	48.00	0.65	15.27	32.12	mrMLM
**AX-91425354**	1	48.00	0.41	8.33	10.85	pLARmEB

*PVE* phenotypic variation explained.

^a^SNPs in bold font are pleiotropic QTNs which were detected to be associated with more than one method.

### QTNs associated with the KMC and KDR

**Figure 3:**
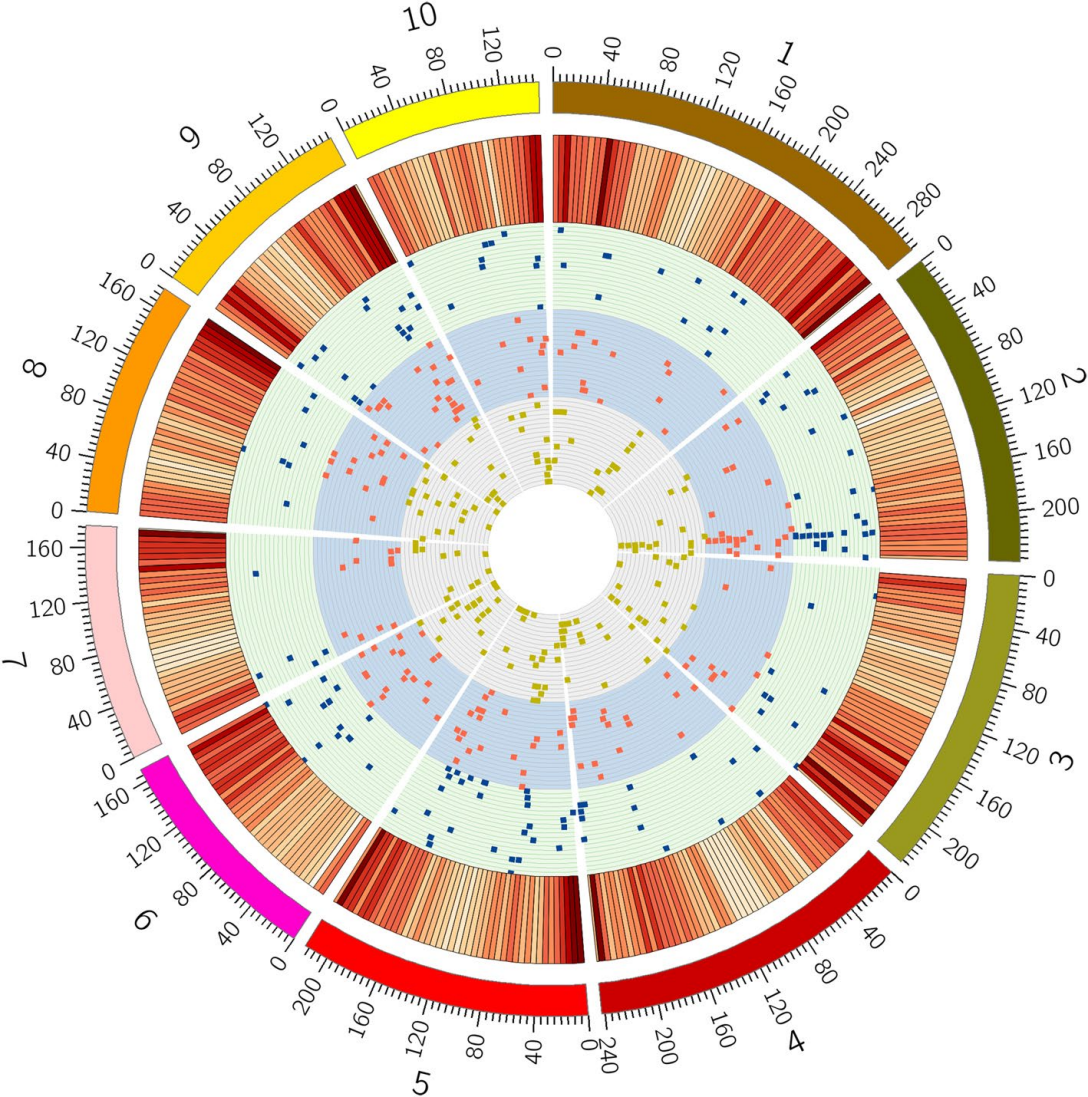
Genome-wide distribution of SNPs throughout the 132 lines’ genomes. The outermost box with the scale represents the 10 maize chromosomes. The red histogram represents the density of SNPs. The yellow, red and blue scatters represent significant QTNs for the KMC and KDR traits in 2014, 2015 and 2016, respectively.

A total of 334 significant QTNs were identified for the KMC and KDR in maize, using five multi-locus GWAS methods in the package mrMLM, with a critical LOD (logarithm of odds) score of 3 (Supplementary Table S1, Fig. 3). A total of 175 significant QTNs were found for the KMC, including 28, 35, 23, 34, 35, 23, and 30 significant QTNs associated with KMC10, KMC15, KMC20, KMC25, KMC30, KMC35, and KMC40, respectively, in which their proportions of phenotypic variation explained (PVE) by each QTN ranged from 1.22 to 25.34%. Of these QTNs, 41 and 21 QTNs were detected in 2 and 3 years, respectively. For the KDR, 178 significant QTNs were detected, including 30 28, 37, 39, 22, and 23 significant QTNs associated with KDR15, KDR20, KDR25, KDR30, KDR35, and KDR40, respectively, in which their PVEs ranged from 0.54 to 24.62%. Among these QTNs, 19 and 5 were detected in 2 and 3 years, respectively. Moreover, 1, 3, 5, and 32 QTNs were found to be simultaneously associated with the above 5, 4, 3 and 2 traits, respectively.

### Common QTNs by multiple methods and years

Among the above-mentioned 334 QTNs, 116 and 58 were detected by two and more than two methods, respectively (Supplementary Tables S2, S3). Nine of the 58 QTNs were detected in 2 years, which were associated with KMC10, KMC20, KMC20, KMC30, KMC35, KMC40, KDR15, KDR30, and KDR40, respectively. Five of the 58 QTNs were detected in 3 years, which were associated with KMC10, KMC30, KMC35, KDR20 and KDR30, respectively. Among the 14 QTNs across multiple environments (Table 3), AX-91654966 on chr.5 was found by five methods in 2015 and 2016 to be associated with KDR40; AX-91629217 on chr.4 was found by four methods in 3 years to be associated with KMC30; and their PVEs ranged up to 19.76% and 14.50%, respectively.

**Table 3:** Fourteen QTNs co-detected by multiple methods and years for KMC and KDR traits.

Trait	SNP	Chr	Pos. (Mb)	QTN effect	LOD score	PVE (%)	No. of methods	Year
KMC10	AX-90785620	2	208.89	− 0.60 ~ − 0.28	3.65 ~ 6.60	3.12 ~ 13.06	3	14, 16
KMC20	AX-123944713	2	210.25	− 0.45 ~ − 0.30	4.04 ~ 6.57	5.81 ~ 13.48	3	14, 15
KMC20	AX-86291963	4	241.80	− 0.67 ~ − 0.27	3.36 ~ 7.67	3.63 ~ 13.3	3	14, 15
KMC30	AX-86286026	1	212.24	− 1.54 ~ − 0.48	4.02 ~ 6.85	2.69 ~ 10.43	3	14, 15
KMC35	AX-90786057	2	210.30	− 2.47 ~ − 1.00	4.15 ~ 6.16	4.18 ~ 9.52	4	15, 16
KMC40	AX-91760347	8	80.68	− 1.82 ~ − 1.49	4.82 ~ 6.97	6.88 ~ 10.01	3	15, 16
KDR15	AX-90848774	3	208.96	0.09 ~ 0.21	3.13 ~ 5.71	3.56 ~ 8.65	3	14, 15
KDR30	AX-91442789	5	71.75	0.11 ~ 0.25	3.76 ~ 10.55	1.83 ~ 13.24	4	15, 16
KDR40	AX-91654966	5	71.07	0.45 ~ 1.13	4.79 ~ 11.6	6.55 ~ 19.76	5	15, 16
KMC10	AX-91785266	9	39.60	− 1.16 ~ − 0.37	3.27 ~ 6.97	4.70 ~ 17.02	3	14, 15, 16
KMC30	AX-91629217	4	173.63	− 1.20 ~ − 0.35	3.17 ~ 9.95	2.76 ~ 14.5	4	14, 15, 16
KMC35	AX-91652225	5	55.51	− 4.23 ~ − 1.55	3.17 ~ 7.93	2.40 ~ 14.34	3	14, 15, 16
KDR20	AX-90843001	5	146.95	0.06 ~ 0.18	3.08 ~ 6.28	0.90 ~ 9.63	3	14, 15, 16
KDR30	AX-90614081	2	237.49	0.09 ~ 0.29	4.12 ~ 11.98	1.30 ~ 15.16	3	14, 15, 16

*PVE* phenotypic variation explained.

### Validation of fourteen common QTNs

**Figure 4:**
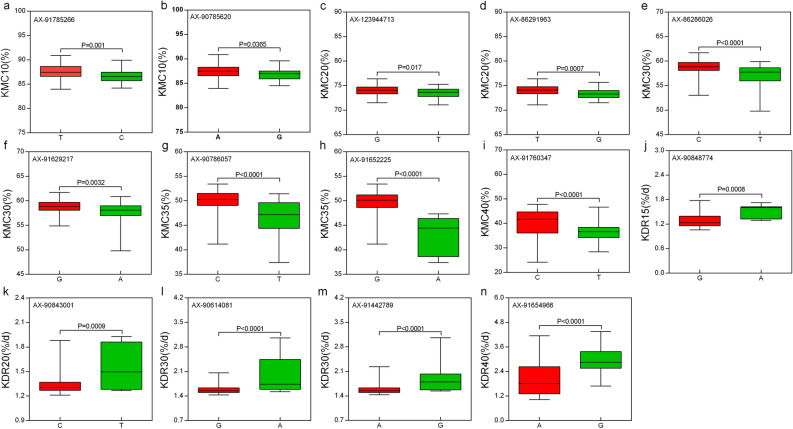
Boxplots for validation of the 14 co-detected QTNs (a ~ n). For each QTN, the population was divided into two groups according to allele types. The X-axis represents the two alleles for each QTN, while the Y-axis corresponds to the phenotype.

Based on the alleles of 14 QTNs, the 132 inbred lines were divided into two groups, and we tested for significant differences in mean phenotypes between the two groups (Fig. 4). The results showed that the mean values of the groups with favourable alleles were larger than those of the other groups with alternative alleles, indicating significant or extremely significant differences.

### GO enrichment analysis

According to the genomic information of B73 Ref Gen_V4, a total of 2,970 genes were present in the intervals adjacent to the 334 QTNs detected for the KMC and KDR in maize (Supplementary Tables S1, S4). A total of 142 genes were detected in the intervals adjacent to the 14 QTNs in two and 3 years. Significance was observed for cellular component in the GO enrichment analysis (P ≤ 0.05; Supplementary Table S5), and this category contained five genes (Supplementary Table S6). This information may be useful for further mining of genes associated with the KMC and KDR in maize.

## Discussion

### Measurements and physiological mechanisms of KMC and KDR

According to Hart and Golumbic^33^, methods to measure seed KMC can be divided into direct and indirect methods. With direct methods, the KMC in a seed sample is measured directly. With indirect methods, the KMC is measured according to the relationship between the chemical and physical characteristics of seed water and its KMC, with calibration to a reference. Direct methods have the advantage of high repeatability, but require a substantial workload in the field and are therefore unsuitable for multiple repeated measurements of many materials. In this study, we directly measured the KMC in maize following the experimental method of Reid et al.^34^. This method can not only reduce the workload in the field but also decrease experimental errors during shelling, thus saving time and labour.

The KMC in maize kernels is closely linked to their dry weight^35^. In the early stage of kernel development, water accumulates more than dry matter. As the dry weight increases, the KMC gradually decreases. Therefore, kernel dehydration before PM is an internal process controlled by growth and development, and environmental factors have no significant effects on dehydration in this process^9^.

Genotypic differences lead to variations in the KDR, and the KDR shows a marked variation between various cultivars before PM, which is still inheritable. This study showed that in the early stage of kernel development, the KMC decreased slowly, while the KDR was relatively low; and with maturation of the kernels, the KMC and KDR increased sharply after 35 DAP (the kernels were close to PM); these trends were the same for 3 years. Although the slope of the KDR or KMC is a parameter used for evaluation of the dehydration process, the index calculated by the fitting curve has no biological meaning. The purpose of the paper is to ensure the key periods and detect the key genes. Meanwhile, the specific moment is also the key point of phenotype identification.

### The feasibility of multi-locus GWAS for detection of QTNs associated with the KMC and KDR

The multi-locus GWAS model has a higher discrimination ability and a lower false-positive rate for detection of animal and plant genes compared with the single-locus GWAS model^36,37^. Geneticists have introduced pleiotropy and population structure into the single-locus GWAS model to reduce the errors in effect estimation by controlling the genetic background^24,38,39^. Although the modification of the single-locus GWAS model improves its detection accuracy to a certain degree, the multiple-testing correction (e.g., Bonferroni correction) of significance thresholds for the single-locus model is too strict. This strict correction leads to the exclusion of important loci, especially when large experimental errors occur in field trials of crop genetics. To solve this problem, the application of multi-locus mrMLM is essential.

Previous studies have applied the multi-locus GWAS model to detect QTNs associated with important traits in various crops. Based on this model, Guan et al. detected 149 QTNs associated with the fatty acid content and composition of rape^40^, while Misra et al. detected 224 significant QTNs for the grain quality of rice, 97 of which were detected by at least two methods^41^. Additionally, Li et al. identified 42 SNPs and 5 QTNs for quality traits of forage sorghum^42^. Peng et al. detected 328 significant QTNs associated with free amino acid (FAA) levels in wheat, including 66 QTNs that were detected by more than two models, which revealed the complexity of metabolic and genetic regulation^43^. Lü et al. detected 159 QTNs for the photosynthetic response of soybean under low-phosphorus stress and discovered 52 candidate genes^44^. Hou et al.^45^ detected 20 QTNs that were significantly associated with drought resistance traits in cotton and further identified 1326 relevant genes, 205 of which were induced after drought stress treatment. Hu et al.^46^ detected 913 QTNs for agronomic traits of barley, including 39 QTNs that were repeatedly detected in various environments and by different methods, with 10 candidate genes identified through gene annotation In maize, Ma et al. ^47^ detected 63 QTNs for the regenerative capacity of embryonic callus and found 40 candidate genes based on these common QTNs. Moreover, Xu et al.^48^ identified a total of 60 QTNs for the gelatinization properties of maize starch. Zhang et al.^49^ detected 423 QTNs for maize stalk traits related to lodging resistance, 29, 34, and 48 of which were associated with stem diameter, stalk bending strength, and rind penetrometer resistance, respectively, as detected by multiple methods or across multiple environments. Based on these studies, conducting multi-locus GWAS for KMC- and KDR-associated traits in maize is feasible.

### Detection of QTLs and QTNs associated with the KMC and KDR

The KMC and KDR of maize are complex quantitative traits with different impact factors in different periods. Physiological dehydration is controlled by genotype and could be stably inherited^11,22,50^. Natural drying is jointly determined by the KMC at PM, drying time, and the KDR and KMC at harvest, which are susceptible to environmental conditions^9^. Both the KMC and KDR have high heritability and can be stably inherited. It is feasible to map major-effect QTLs for the KMC and KDR, to mine associated candidate genes, and to develop practical functional markers for marker-assisted selection, which will be valuable for the breeding of maize cultivars for rapid dehydration. Many studies have mapped QTLs for the KMC and the KDR in maize^3,4,16–19^, but GWAS for these two traits have rarely been reported. Dai et al.^20^ selected 80 maize inbred lines to conduct a field survey for two consecutive years, performed a GWAS using 1,490,007 high-quality SNPs, and eventually detected 19 SNPs associated with natural KDR in the field. Zhang et al. conducted GWAS on kernel dehydration traits in maize using 290 inbred lines in combination with 201 simple sequence repeat (SSR) molecular markers, and 17 SSRs associated with natural KDR in the field were finally detected^21^. This study conducted a multi-locus GWAS for KMC- and KDR-associated traits in maize based on the Axiom Maize 55K Array data of 132 maize inbred lines. A total of 116 and 58 QTNs were detected by two and more than two methods, respectively. Among the 58 QTNs detected by three or more methods, 9 and 5 were detected in 2 or 3 years.

The QTNs identified in this study were correlated with QTLs reported earlier than the present report. Among these 14 QTNs, AX-123944713 and AX-90786057 were located in the *qKdr-2-1* interval detected by Wang et al.^51^ and the intervals of *q9GDR13-2-1* and *qcGDR23-2-1* by Li et al.^18^; AX-90614081 were located in the *q8GDR14-2-1* and *qcGDR14-2-2* interval detected by Li et al.^18^ and the *qKdr-2-2* interval by Liu et al.^19^; AX-90843001 was located in the intervals of *q8GWC20-5-1*, *q9GWC10-5-2*, *qcGWC10-5-1*, *qcGWC20-5-1*, *q8GDR12-5-1* and *qcGDR12-5-1* detected by Li et al.^18^; AX-90848774 was located in the *qKdr-3-6* interval detected by Wang et al.^51^; AX-91652225 was located in the *q9GWC10-5-11* interval detected by Li et al.^18^; AX-91654966 and AX-91442789 were located in the intervals of *q8GWC10-5-1*, *q8GWC30-5-1*, *q8GWC40-5-1*, *q9GWC20-5-1*, *q9GWC30-5-1*, *q9GWC40-5-1*, *qcGWC30-5-1*, *qcGWC40-5-1*, *q8GDR13-5-1*, *q8GDR14-5-1* and *qcGDR13-5-1* detected by Li et al.^18^; AX-91760347 was located in *qKdr-8-2* interval detected by Wang et al.^51^; AX-91785266 was located in intervals of *q9GWC30-9-1*、*q9GDR13-9-1*、*q9GDR34-9-1* and *qcGDR13-9-1* detected by Li et al.^18^; AX-86286026, AX-91629217 and AX-86291963 have not been reported. Furthermore, a significant enrichment in cellular component was obtained through GO enrichment analysis of candidate genes in the intervals adjacent to the 14 QTNs detected in 2 or 3 years, and this category contained five genes.

## Conclusions

The 132 inbred lines were genotyped using a maize 55K single-nucleotide polymorphism array. QTNs for the KMC and KDR in maize were detected based on five methods (mrMLM, FASTmrMLM, FASTmrEMMA, pLARmEB, and ISIS EM-BLASSO) in the package mrMLM. A total of 334 significant QTNs were identified for the KMC and the KDR, 116 and 58 of which were detected by two and more than two methods, respectively, while 9 and 5 QTNs were detected in 2 and 3 years, respectively. A significant enrichment in cellular component was revealed by GO enrichment analysis of candidate genes in the intervals adjacent to the fourteen QTNs, and this category contained five genes. The information provided in this study may be useful for further mining of genes associated with the KMC and the KDR in maize.

## Materials and methods

### Plant materials

A total of 132 maize inbred lines were used in this study, which were provided by the Institute of Crop Resources, Jilin Academy of Agricultural Sciences (JAAS) (Supplementary Table S7). These lines represented six subpopulations: BSSS (American BSSS including Reid), PA (group A germplasm derived from modern U.S. hybrids in China), PB (group B germplasm derived from modern U.S. hybrids in China), Lan (Lancaster Surecrop), LRC (derivative lines from Lvda Red Cob, a Chinese landrace), and SPT (derivative lines from Si-ping-tou, a Chinese landrace).

### Field design and phenotypic measurement

The 132 lines were grown at the Gongzhuling (124°47′ N and 43°27′ E, Jilin Province, China) Experimental Base, JAAS in 2014, 2015 and 2016, with a final plant density of 75,000 plants ha^-1^. A randomized block experimental design with three replications was adopted. In 3 years, each plot had 5 rows, with a row length of 5 m, row spacing of 0.65 m, plant spacing of 0.20 m and plot area of 16.25 m^2^. Normal agronomic practices for maize were used in field management. To avoid border effects, for each plot, two border rows and the first two plants at each end of the middle three rows were not used for future trait determination.

The ears were bagged before silking (50% of plants in the row with extruded silks). Then the bagged ears were pollinated by hand. One week later, the bags were removed and five tested ears were randomly selected, tagged and labelled in each plot. The moisture concentration was recorded from 10 to 40 DAP, with one measurement of every five days. At 9:00 a.m., per the method published by Reid et al.^34^, for each ear, a SK-300 probe for moisture concentration measurement (manufactured by Harbin Yuda Electronic Technology Co., Ltd., China) was used to pierce into kernels after penetrating the bract leaves in the middle of the ear.

KMCs at 10, 15, 20, 25, 30, 35, and 40 DAP were measured, which were designated as KMC10, KMC15, KMC20, KMC25, KMC30, KMC35, and KMC40, respectively. KDRs were then calculated based on two consecutive KMC measurements. KDR = (KMC at a given time—KMC at the next time)/number of days during the time span. The KDRs for the six time spans (namely, 10–15, 15–20, 20–25, 25–30, 30–35 and 35–40 DAP) were denoted as KDR15, KDR20, KDR25, KDR30, KDR35, and KDR40, respectively.

### Statistical analysis of phenotypic data

The F-values for genotypes (F_G_) and environments phenotypic data (F_E_) were analysed by SPSS 22 (IBM Corp., Armonk, NY, USA). Broad-sense heritability was calculated using the formula proposed by Knapp et al.^52^: ${H}^{2}={\delta }_{g}^{2}/\left[{\delta }_{g}^{2}+\left({\delta }_{gl}^{2}/n\right)+{\delta }_{e}^{2}/nr\right]$, where ${\delta }_{g}^{2}$ is the genetic variance, ${\delta }_{gl}^{2}$ is the variance of the genotype-by-environment interaction, ${\delta }_{e}^{2}$ is the error variance, n is the number of sites, and r is the number of replicates.$CV=SD/Mean$.

### Genotyping and filtering

Genomic DNA was extracted from young leaf samples of the 132 maize inbred lines using the modified cetyltrimethylammonium bromide (CTAB) method^53^. The quality of DNA was assessed using 0.8% agarose gel electrophoresis and a NanoDrop 2000 spectrophotometer (NanoDrop, Wilmington, DE, USA). Genotyping was performed using the Axiom Maize 55K SNP Array (CapitalBio Corp., Beijing, China) ^31^, which contained a total of 55,229 SNPs. The 132 inbred lines were genotyped based on the Affy AXIOM Array 2.0 platform. Genotyping data of the 132 inbred lines were filtered using the software PLINK^54^ with the settings of MAF > 0.05, GENO < 0.1, and genotype heterozygous loci missing.

### Linkage disequilibrium and population structure analysis

LD among markers was calculated using PLINK software. The window size for LD calculation was set based on the number of SNPs located in the genome. Pair-wise linkage disequilibrium was measured using the squared allele frequency correlations, according to Weir^55^, and assessed by calculating r^2^ for pairs of SNP loci.

The population structure of 132 lines was assessed using STRUCTURE software 2.3.4^56^. A burn-in of 5000 iterations followed by 50 000 Markov Chain Monte Carlo (MCMC) replicates was implemented to estimate the number of subpopulations (*k*) in a putative range of 2–10. To estimate the robustness of the inferred population structure, five replications were conducted for each k. The subpopulation number was estimated using an ad hoc statistic delta k based on the rate of change in the log probability of data between successive values^57^.

### Genome-wide association studies

All the phenotypic and genotypic information in the above mapping population was used to detect QTNs using the mrMLM^26^, FASTmrEMMA^29^, FASTmrMLM^30^, pLARmEB^28^, and ISIS EM-BLASSO^27^ approaches, implemented by the software programme mrMLM v4.0 (https://cran.r-project.org/web/packages/mrMLM.GUI/index.html). The unified parameter settings for the five methods were as follows: (1) the Q + K model was used, where the population structure value Q was calculated by Structure 2.3.4 software^56^, and the kinship value K was analyzed by the “mrMLM” software package; and (2) the significance threshold *p* value was set as 0.0002 (limit of detection (LOD) = 3.0). In addition, while using mrMLM and FASTmrEMMA, the search radius of candidate genes was specified as 20 kb; using pLARmEB, 50 potential association loci were selected on each chromosome.

### Annotation of candidate genes analysis

QTNs for the KMC and the KDR in maize detected by multiple methods were mapped to the maize reference genome B73 RefGen_V4 to identify associated candidate genes. The obtained candidate genes were subjected to GO enrichment analysis using AgriGO v2.0^58^, and the final set of genes associated with the KMC and the KDR were identified.

## Supplementary Information

Supplementary Table S1.

Supplementary Table S2.

Supplementary Table S3.

Supplementary Table S4.

Supplementary Table S5.

Supplementary Table S6.

Supplementary Table S7.
